# Bloodless Management of Severe Refractory ITP and Acute Hemorrhage in a Jehovah's Witness Patient

**DOI:** 10.1002/ccr3.70102

**Published:** 2025-01-29

**Authors:** Gagandeep Kaur, Shanley Banaag, Lee Hong, Charisma Mylavarapu, Yuri Kim, John Garrett, Emily Nagler

**Affiliations:** ^1^ Scripps Mercy Hospital San Diego California USA; ^2^ Scripps Clinic Medical Group San Diego California USA; ^3^ Moores Cancer Center University of California San Diego San Diego California USA

**Keywords:** bloodless management, erythropoietin, hemorrhage, immune thrombocytopenia, Jehovah's Witness

## Abstract

In patients with severe refractory immune thrombocytopenia (ITP), especially those unable to receive blood transfusions due to religious beliefs, alternative non‐cytotoxic therapies are important to avoid worsening cytopenias. Immunomodulatory agents such as mycophenolate mofetil and daratumumab should be used alongside traditional therapies including steroids, IVIG and rituximab.

## 1 Introduction

Thrombocytopenia is defined as a platelet count of less than 150 × 10^3^/μL. The primary mechanisms leading to a reduced platelet count include decreased production, increased platelet destruction, platelet sequestration, and hemodilution [1]. Immune thrombocytopenia (ITP) is an autoimmune disorder characterized by isolated thrombocytopenia, with a prevalence of 10 per 100,000 individuals, and is primarily driven by increased platelet destruction [2, 3]. The key pathogenesis is incompletely understood but is thought to involve anti‐platelet IgG antibodies which bind to platelet glycoproteins GPαIIbβ3 (GPIIb/IIIA) and GPIb‐IX‐V and subsequently label them for peripheral destruction in the liver and spleen [4, 5]. Autoreactive cytotoxic CD8+ T cells and the cell‐mediated lysis of platelets may also play a role in the pathogenesis [6]. Patients may be asymptomatic or present with bruising, petechiae, or, in rare cases, life‐threatening bleeding [7]. ITP is a diagnosis of exclusion so other potential causes of thrombocytopenia, such as infections, malignancies, and medications should be investigated [8, 9]. A clinical history of isolated thrombocytopenia with improvement following treatment with steroids and intravenous immunoglobulin (IVIG) is highly suggestive of ITP.

While mild to moderate cases of ITP are often managed in the outpatient setting, severe cases (platelet count < 20 × 10^3^/μL) typically require inpatient care due to the heightened risk of spontaneous bleeding and the need for close monitoring of treatment efficacy via daily laboratory assessment. Steroids are the first line of therapy in ITP and complete remission can be seen in 60%–80% of patients with oral prednisone in the outpatient setting [10, 11]. Despite these high response rates, the majority of patients experience disease relapse, with approximately 60%–70% progressing to persistent or chronic ITP [12]. In patients refractory to steroids, rituximab, TPO‐RAs, and splenectomy can be used as second‐line therapy with splenectomy being the best curative therapy [13]. Managing bleeding and proceeding to splenectomy in patients with severe thrombocytopenia and hemorrhagic complications can be difficult especially in those unable to receive any blood products. In addition to bleeding complications, ITP presents several challenges, including the disease's unpredictability [14], the need for different treatment modalities, and the importance of regular outpatient follow‐up. Here we describe the bloodless management of a 50‐year‐old female Jehovah's Witness patient who presented with severe thrombocytopenia in the setting of recent tooth infection and antibiotic use. This case aims to highlight the efficacy of multimodal bloodless management of severe refractory ITP and blood loss anemia in Jehovah's Witness individuals who decline blood products.

## 2 Case History/Examination

A 50‐year‐old female presented to the emergency department with a chief complaint of generalized body rash on her extremities (“spots”), headache, gingival bleeding, and small‐volume epistaxis 1 week before presentation. She has had a chronic dental infection requiring multiple antibiotics, initially treated with amoxicillin and subsequently switched to cephalexin about 1 week prior to presentation. Around this time, she noted that she was developing “spots” all over her body, more prominently on her arms and legs, bleeding with brushing her teeth and occasional small‐volume nosebleeds. Social history was notable for the patient was studying to be a Jehovah's Witness and she declined all blood products due to her religious beliefs.

Vital signs in the emergency department were overall unremarkable with no evidence of hemodynamic instability (temperature 36.8°C, pulse 61, respiratory rate 22, SpO_2_ 98% on room air, blood pressure 127/81). Physical exam was significant for wet purpura with scattered petechiae on bilateral upper and lower extremities. Laboratory findings were significant for new anemia with hemoglobin of 11.1 g/dL, MCV 93 μm^3^, RDW 14.3%, and platelets of less than 2000/mm^3^. Five months prior to presentation, her hemoglobin and platelets were within normal limits (12.4 g/dL and 291,000/mm^3^, respectively). INR 1.0. Imaging was pertinent for no splenomegaly on abdominal ultrasound and CT head was negative for acute intracranial process. Peripheral smear revealed no schistocytes; bone marrow biopsy was negative for an infiltrative process.

## 3 Differential Diagnosis, Investigations and Treatment

She was started on dexamethasone and intravenous immunoglobulin (IVIG; see Table 1), but due to persistent thrombocytopenia was switched to rituximab. Eltrombopag was added on hospital day 9 then switched to avatrombopag on day 24, as it is a newer‐generation thrombopoietin receptor agonist (TPO‐RA) which may have better efficacy compared to older‐generation TPO‐RAs [17]. Due to persistent thrombocytopenia, the treatment team considered splenectomy but unfortunately she was deemed a poor surgical candidate for splenectomy given the lack of response to medical therapy and significant bleeding risk.

**TABLE 1:** Summary of medications used for immune thrombocytopenia.

Medication	Expected median onset of action for ITP	Dosage	Timeline of administration in hospital
Dexamethasone	2–14 days	40 mg IV × 3 20 mg IV × 4	Week 1–4 and 11–14
		10 mg IV × 3	
Intravenous Immunoglobulin	1–3 days	1 g/kg IV × 3	Week 1 and 10
		0.5 g/kg IV × 1	
Rituximab	7–56 days	375 mg/m^2^ IV weekly × 4	Week 1–4
Eltrombopag	1–2 weeks	50 mg PO QD × 14 days	Week 2–4
Avatrombopag	3–5 days	20 mg PO QD × 14 days	Week 4–8
		40 mg PO QD × 8 days	
Fostamatinib	15 days	100 mg PO BID × 9 days	Week 7–14
		100 mg PO TID × 16 days	
		150 mg PO BID × 37 days	
Daratumumab	6 weeks [15]	16 mg/kg IV weekly × 4	Week 11–14
Mycophenolate mofetil	4–6 weeks [16]	500 mg PO BID × 11 days	Week 11–12

The patient's hospitalization was complicated by anemia due to epistaxis, hematuria, and intermittent oral, vaginal, and rectal bleeding. She was treated with iron sucrose and epoetin alfa. On hospital day 45, she had a witnessed fall with no head trauma and subsequent bilateral conjunctival hemorrhages. CT head revealed subarachnoid and left parietal lobe hemorrhage. She was started on tranexamic acid (TXA) and transferred to ICU for closer monitoring (received 12 g total). She was transferred from the ICU on hospital day 48 given stable neurological status. Even with resuming avatrombopag, the patient demonstrated progressing confusion and repeat CT head demonstrated interval increase in the size of intracranial hemorrhage and persistently undetectable platelet levels.

She developed an acute DVT on day 55, at which point avatrombopag and TXA were held, and the patient was started on fostamatinib. Given the ongoing severity of her ITP and concern for hemorrhage with splenectomy, she consented to low‐dose splenic radiation with splenectomy vaccinations prior as previously described [18]. Her fostamatinib dosage was increased and IVIG was reintroduced given no response to fostamatinib for more than 14 days.

## 4 Conclusion and Results (Outcome and Follow‐Up)

**FIGURE 1:**
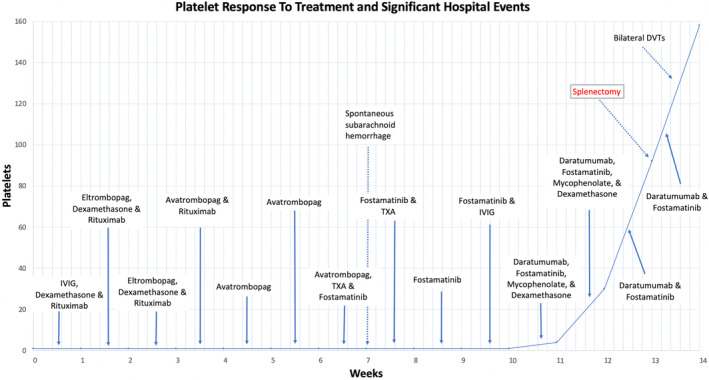
Platelet count over time during hospitalization. Medications administered over time are labeled with arrows and significant events are labeled with dashed arrows.

Weekly daratumumab and mycophenolate mofetil were started on day 74 which finally improved platelets to 18,000/mm^3^ however was complicated by mycophenolate‐induced leukopenia which resolved following discontinuation. Platelets continued to improve, and the patient underwent splenic artery embolization and laparoscopic splenectomy on hospital day 92. Postop course was complicated by bilateral lower extremity DVTs treated with heparin drip then apixaban. On discharge from hospital, platelets were 158,000/mm^3^ and patient was given fostamatinib for two more days pending outpatient hematology follow‐up (Figure 1).

## 5 Discussion

One of the major obstacles in managing this patient was supporting hemoglobin and platelet levels without transfusions (bloodless management). Though specific strategies may vary, retrospective cohort trials demonstrated bloodless management primarily in the setting of cardiac surgery is associated with similar outcomes in morbidity and mortality compared to conventional management [19, 20]. We will review the mechanisms of action and rationale for usage of TPO‐RAs, erythropoietin stimulating agents (ESAs), TXA, and other novel agents for ITP and acute hemorrhage in bloodless management (doses summarized in Table 1).

TPO‐RAs have shifted the paradigm of ITP management [21]. TPO‐RAs bind to the TPO receptor leading to a conformation change in the receptor and stimulation of the JAK2‐STAT5 pathway culminating in megakaryocyte proliferation and platelet production. Eltrombopag, a small hydrazone molecule, and avatrombopag, another small molecule, bind to the transmembrane portion of the TPO receptor [22]. Studies have shown an overall response rate of 75% and a durable response rate at 6 months of 65% with TPO‐Ras [23, 24]. For patients who fail to respond to treatment with one TPO‐RA, switching to another has yielded a response in 50%–80% of patients [23, 25]. TPO‐RAs are approved second‐line therapies for ITP demonstrating significantly improved platelet counts and complete response in the chronic ITP setting [26]. TPO‐RAs and ESAs are accepted forms of treatment in the Jehovah's Witness community [27]. We used TPO‐RAs in standard protocol for refractory ITP management, escalating doses of a single TPO‐RA before trial with alternative TPO‐RA. ESAs are frequently utilized to manage anemia in bloodless management. These agents increase erythroid precursor production in the bone marrow, typically combined in most protocols with intravenous iron, vitamin B12, and folate supplementation [26, 27, 28].

Given the patient's refusal of red blood cell transfusions and the presence of severe ongoing anemia, we determined that traditional cytotoxic regimens for refractory ITP—including high‐dose cyclophosphamide, 6‐mercaptopurine, azathioprine, and vinca alkaloids—were not suitable options due to the risk of worsening anemia [29]. We next turned to other newer and off‐label medications for refractory ITP. Fostamatinib, an oral spleen tyrosine kinase (SYK) inhibitor, is approved for refractory ITP with median response duration of about 15 days [30]. Although fostamatinib initially appeared to be a promising therapy for patients with refractory ITP based on meta‐analysis and with 85.4% response rates in clinical practice as seen in Spanish centers, real‐world evidence has been mixed and unfortunately did not lead to significant elevations in platelet count for our patient [31, 32]. Mycophenolate modefil, which directly inhibits T cells, has also been shown to induce complete responses when used as a second‐line or greater steroid‐sparing agent given at 1 g/day [33]. Daratumumab, an anti‐CD38 antibody widely used in multiple myeloma, has previously been shown to improve autoimmune cytopenias [34] and can induce long‐term remission for ITP refractory to splenectomy and corticosteroids [35]. By targeting long‐lived plasma cells that are classically CD20‐negative, we hypothesized that daratumumab might be able to eliminate a unique population of antiplatelet‐producing cells that are preserved after rituximab therapy as previously described [36]. We administered 16 mg/kg IV on days 1, 8, 15, and 22 for one cycle after patient consented to off‐label use and continued fostamatinib and mycophenolate as above. Approximately 1 week after starting daratumumab and 4 days after starting mycophenolate modefil, the patient's platelet count began to rise exponentially which is likely from the effects of prior combination therapy (rituximab, TPO‐RAs, and fostamatinib) and the immediate effects of daratumumab and mycophenolate modefil. In hindsight, an alternative strategy for managing this patient's refractory ITP could have implemented immunomodulators earlier in lines of therapy and hopefully could have prevented episodes of acute blood loss anemia.

As our patient was having life‐threatening refractory bleeding from intracranial hemorrhage, we added TXA to her treatment. TXA is a synthetic lysine amino acid derivative with antifibrinolytic effects by inhibiting lysine receptors on plasminogen stabilizing the fibrin's matrix structure. There have been multiple case series on the use of TXA in 12 patients with ITP [15]. All patients utilized TXA dosages ranging from 0.5 to 3 g/day and did not need transfusions of platelets or red blood cells. Another case report using TXA in life‐threatening nontraumatic hemorrhage ITP showed that IV TXA and transfusions resolved a pediatric patient's episode of significant bleeding and hypotension [37]. No adverse events from TXA were noted [15, 37].

TXA is used in many bleeding disorders, with benefits in reducing rebleeding risk with oral or low‐dose IV TXA without the thrombotic side effects [38]. It is often taught that TXA should be withheld in the setting of hematuria given risk of renal failure secondary to thrombotic obstruction of ureter. However, this has been based on low quality evidence. A review of three randomized control trials and three retrospective cohort studies by Lee et al. [39] involved 466 patients receiving TXA, 342 of whom had hematuria and received TXA, only 28 documented renal function which did not worsen with TXA use.

Our patient developed bilateral DVTs shortly after her splenectomy. Previous studies have demonstrated that patients with ITP who undergo splenectomy face a higher risk of sepsis and venous thromboembolism compared to those who do not [40, 41, 42]. Although our patient responded well to pharmacological treatments, we recommended splenic embolization followed by a splenectomy before hospital discharge. This decision was based on concerns regarding outpatient follow‐up due to transportation challenges. We believed that the benefits of a more definitive treatment outweighed the associated risks.

In conclusion, this case highlights the multimodal bloodless management of severe refractory ITP and acute hemorrhagic anemia in a Jehovah's Witness patient. To avoid cytotoxic regimens for ITP, we used alternative approaches with immunomodulatory therapies to achieve treatment response. Her acute hemorrhage and anemia were managed with TXA, iron supplementation, and ESA. She underwent a splenectomy for definitive treatment of her ITP. Immunomodulatory therapies should be considered in patients with refractory ITP.

## Author Contributions

G.K., S.B., L.H., Y.K., C.M. all wrote the manuscript with equal contributions. E.N. and J.G. helped with conception, writing and editing the manuscript.

## Consent

Patient consent was obtained with written informed consent to publish this report in accordance with the journal's patient consent policy.

## Conflicts of Interest

E.N. served on advisory boards for Protagonist, Sobi, and Pharmaessentia.

## Data Availability

Data sharing is not applicable to this article as no new data were created or analyzed in this study.
